# Peripheral Inflammation Results in Increased Excitability of Capsaicin-Insensitive Nociceptive DRG Neurons Mediated by Upregulation of ASICs and Voltage-Gated Ion Channels

**DOI:** 10.3389/fncel.2021.723295

**Published:** 2021-10-18

**Authors:** Dmytro E. Duzhyy, Nana V. Voitenko, Pavel V. Belan

**Affiliations:** ^1^Department of Sensory Signaling, Bogomoletz Institute of Physiology, Kyiv, Ukraine; ^2^Department of Molecular Physiology and Biophysics, Kyiv Academic University, Kyiv, Ukraine; ^3^Research Center, Dobrobut Academy, Kyiv, Ukraine; ^4^Department of Molecular Biophysics, Bogomoletz Institute of Physiology, Kyiv, Ukraine

**Keywords:** pain, inflammation, capsaicin, DRG neuron, acid-sensing ion channel (ASIC), T-type channels, excitability, spontaneous activity

## Abstract

Previously, we have characterized the capsaicin-insensitive low pH-sensitive (caps^−^lpH^+^) subtype of small-sized nociceptive dorsal root ganglion (DRG) neurons that express acid-sensing ion channels, T-type Ca^2+^ channels, and have isolectin B4-negative phenotype. These neurons demonstrated increased excitability in a model of long-term diabetes, contributing to chronic pain sensation. Here we studied changes in the excitability of the caps^−^lpH^+^ neurons and underlying changes in the functional expression and gating properties of ion channels under complete Freund's adjuvant (CFA)-induced peripheral inflammation. We have found that, under these pathological conditions, the functional expression of the acid-sensing ion channels (ASICs) and voltage-gated Na^+^ channels, was increased. In addition, T-type Ca^2+^ current was significantly increased in the neurons at the membrane potentials close to its resting value. Altogether, the observed changes in the channel functioning shifted a pH level evoking an action potential (AP) toward its physiological value and led to an increase of evoked and spontaneous excitability of the caps^−^lpH^+^ neurons that may contribute to hyperalgesia and chronic inflammatory pain.

## Introduction

Under inflammation the nociceptive dorsal root ganglion (DRG) neurons become increasingly excited by innocuous stimulus or spontaneously active, opening the gate for maladaptive pain (Woolf and Ma, 2007; Costigan et al., 2009). Such sensitization is caused by the changes in the expression and regulation of ligand- and voltage-gated ion channels (Woolf and Costigan, 1999; Campbell and Meyer, 2006; Woolf and Ma, 2007). In particular, transient receptor potential (TRP) (Obata et al., 2005; Ikeda-Miyagawa et al., 2015), P2X (Xu and Huang, 2002), acid-sensing ion channel (ASIC) (Voilley et al., 2001), Na^+^ (Gould et al., 1998; Tanaka et al., 1998), and T-type Ca^2+^ channels (Huang et al., 2016) become increasingly expressed in the small-sized nociceptive DRG neurons.

The population of small-sized nociceptive DRG neurons encompasses several subtypes with the different sensory modalities and sets of voltage-gated K^+^, Na^+^, and Ca^2+^ channels, which endue them with different excitability characteristics (Petruska et al., 2000). Previously, using the protocols described by Petruska and colleagues (Petruska et al., 2000), we isolated the capsaicin-insensitive low pH-sensitive (caps^−^lpH^+^) subtype of small-sized nociceptive DRG neurons that demonstrated increased excitability under streptozotocin (STZ)-induced diabetes due to upregulation of the T-type Ca^2+^ channels (Duzhyy et al., 2015). Taking into account that inflammation is one of the pathogenic complications of diabetes (Feldman, 2003; Shanmugam et al., 2003) and the previous findings of other researchers about changes in the expression of ASIC (Voilley et al., 2001; Mamet et al., 2002), Na^+^ (Gould et al., 1998; Tanaka et al., 1998), and T-type Ca^2+^ channels (Huang et al., 2016) in small-sized nociceptive DRG neurons under inflammatory conditions, we have suggested that the caps^−^lpH^+^ DRG neurons should also demonstrate increased excitability under these conditions. In this study, we aimed to explore the changes in the excitability of caps^−^lpH^+^ DRG neurons under complete Freund's adjuvant (CFA)-induced peripheral inflammation and underlying changes in the functional expression and gating properties of ASICs and voltage-gated Na^+^, K^+^, and T-type Ca^2+^ channels.

## Materials and Methods

All animal care and handling were done in accordance with the protocols of the Animal Care and Use Committee at the Bogomoletz Institute of Physiology, Ukraine, and conformed to the NIH Guide for the Care and Use of Laboratory Animals and the Public Health Policy. An approval protocol of the Bioethics Committee of Bogomoletz Institute of Physiology No 3/19 from 04/02/2019.

### Induction of Peripheral Inflammation

Peripheral inflammation was induced by the subcutaneous injection of 100–150 μl of CFA from *Mycobacterium tuberculosis*, suspended in an oil-saline (1:1) emulsion, into a plantar side of hind paws of male Wistar rats weighing 200 ± 20 g. An oil-saline (1:1) emulsion without CFA was used to inject the paws of control rats. The development of the inflammation was controlled visually, by edema of the paw, and in the behavioral tests based on the development of inflammatory-induced thermal and mechanical hyperalgesia, as routinely performed in our laboratory (Kopach et al., 2012, 2013).

### Acutely Dissociated DRG Neurons

Capsaicin-insensitive low pH-sensitive DRG neurons were acutely isolated from lumbar L_4_-L_6_ DRG of CFA- and saline-injected rats on a second day after injections, and identified as described previously (Duzhyy et al., 2015).

### Electrophysiology

According to Petruska et al. (2000) and Duzhyy et al. (2015), the subtypes of DRG neurons can be unequivocally identified using three voltage clamp protocols of the current activation giving particular current signatures, characterized by the activation thresholds and kinetics of activation and inactivation of the total current. Earlier we confirmed that the caps^−^lpH^+^ neurons (based on challenging with capsaicin, low pH, and IB4 staining) had a particular current signature in these protocols (Duzhyy et al., 2015). In the current work, these protocols were used to identify the caps^−^lpH^+^ DRG neurons. Whole-cell electrophysiological recordings were performed as described previously (Duzhyy et al., 2015) except for the following differences. The same internal solution was used for determining the subtype of neurons, recording Ba^2+^, Na^+^, and K^+^ currents, and for all the current-clamp experiments. This KCl–KMethanesulfonate solution contained the following (in mM): 10 KCl, 135 methanesulfonic acid, 4 MgATP, 0.4 NaGTP, 5 Na_2_-phosphocreatine, 10 N-(2-hydroxyethyl)piperazine-N′ 2-(2-ethanesulfonic acid) (HEPES), 0.2 ethylene glycol-bis(2-aminoethylether)-N,N,N′,N′-tetraacetic acid (EGTA), pH 7.3 with KOH, osmolarity 296 mOsm. The external solution used for the recordings of Na^+^, K^+^, and ASIC currents was Tyrode's solution containing (in mM): 140 NaCl, 4 KCl, 2 MgCl_2_, 2 CaCl_2_, 10 glucose, and 10 HEPES, adjusted to pH 7.4 with NaOH. An activation protocol for recording the total voltage-gated current included depolarization steps from a holding potential of −100 mV (3.5 s) to the test potentials from −60 to +40 mV (200 ms) with a 20 mV increments. The K^+^ current value was measured at the end of depolarization step when Na^+^ current was almost inactivated while the contribution of Ca^2+^ current was negligible. A transient Ba^2+^ current *via* T-type Ca^2+^ channels (T-type current) (Duzhyy et al., 2015) was recorded using an Ba^2+^-TEA-Cl-based external solution containing (in mM): 165 tetraethylammonium (TEA)-Cl, 10 HEPES, 2 BaCl_2_, pH 7.4 with TEA-OH, osmolarity 305–315 mOsm. Its amplitude was measured as a difference between a value of current at the peak and at the end of depolarization step. For recordings of the T-type current at the lower pH values, ranging from 6.5 to 7.4, pH of TEA-Cl-based solution was adjusted to a required value by decreasing an amount of added TEA-OH. A liquid junction potential between an internal solution with KCl-KMethanesulfonate and external Tyrode's solution was 9.7 mV while it was 15.4 mV between the same internal solution and TEA-Cl based external solution. The junction potentials were calculated using an online program LJCalc (https://swharden.com/software/LJPcalc/) and were not compensated.

An activation protocol for recording T-type current consisted of the depolarization steps from a holding potential of −100 mV (3.5 s) to the test potentials from −80 to 0 mV (250 ms) with 10 mV increment, while a steady-state inactivation protocol consisted of the depolarization steps to a test potential of −40 mV (250 ms) from a holding potential ranging from −100 to −50 mV (3.5 s) with 10 mV increment.

The protocol for the action potentials (APs) generation consisted of a series of increasing current injections (350 ms long, with 5 s intervals between the injections) evoking incremental depolarization (2–3 mV per step) from a holding potential of −90 mV.

Acid-sensing ion channel currents were evoked in neurons by a fast application of low pH Tyrode's solution using Quartz MicroManifold (ALA Scientific, NY, USA) and recorded in a voltage-clamp mode at a holding potential of −60 mV. Tyrode's solution was buffered with HEPES in the pH range 6.5–7.4 and with 2-(N-morpholino)ethanesulfonic (MES) at pH lower than 6.5; pH was adjusted to a required value with NaOH. The evoking APs by an application of low pH Tyrode's solution was performed in a current clamp mode at a membrane potential of −60 mV.

### Analysis

**Figure 1:**
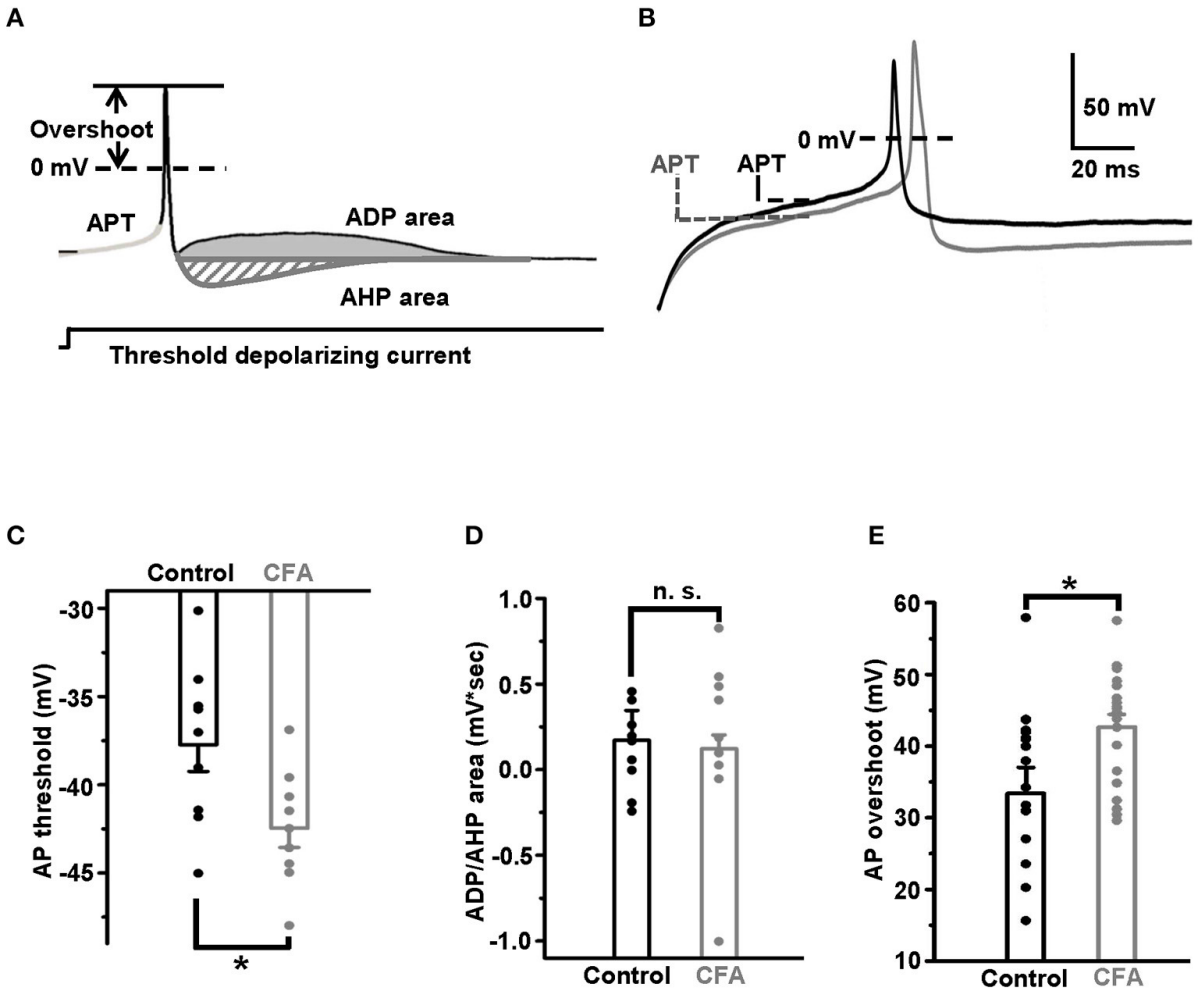
Excitability of the capsaicin-insensitive low pH-sensitive (caps^−^lpH^+^) neurons is increased under the complete Freund's adjuvant (CFA)-induced inflammation. **(A)** The parameters of action potential used for estimating changes in excitability of the neurons: action potential threshold (APT, mV), afterdepolarization/hyperpolarization potential (ADP/AHP) areas (mV*s) and action potential (AP) overshoot (mV). The parameters were obtained for APs evoked in the response to a threshold depolarizing current pulse. A threshold depolarizing current pulse is shown below an AP. **(B)** The representative traces of threshold APs demonstrate a decrease of APT and increase of AP overshoot in the neurons from rats with inflammation (gray) compared with control (black). **(C)** Pooled results show a significant decrease of APT under the CFA-induced inflammation. **(D)** No significant change in the ADP/AHP area was observed under the CFA-induced inflammation. **(E)** A significant increase of AP overshoot under the CFA-induced inflammation. ^*^p < 0.05.

Three parameters of APs were used to compare the excitability of DRG neurons in the normal conditions and under CFA-induced inflammation. All of them were measured at a minimal (threshold) current stimulation sufficient for an AP generation. The first parameter, the AP threshold, was defined as a membrane potential at an inflection point preceding an AP upstroke (Duzhyy et al., 2015) (Figure 1A). The second parameter, the AP overshoot, was determined as a maximal value of AP exceeding 0 mV (Figure 1A). The third excitability parameter used in this study was defined previously as an area (mV^*^s) between the stationary level of recorded potential, determined at the end of the current injection step, and the afterdepolarization potential (ADP) or the afterhyperpolarization potential (AHP) determined at a threshold current injection evoking an AP (Duzhyy et al., 2015) (Figure 1A). The higher ADP value was shown to correlate with the higher probability of AP burst generation (Duzhyy et al., 2015).

The ion conductance *G(V)* and a current density during a steady-state inactivation protocol *I(V)* were calculated as previously described (Duzhyy et al., 2015) and fitted with the following Boltzmann functions:


(1) $$\begin{array}{l} G\left(V\right)=\frac{G_{\max}}{\left(1+e^{\left(\frac{\left(V-V_{50}\right)}{k}\right)}\right)} \end{array}$$

and


(2) $$\begin{array}{l} I\left(V\right)=\frac{I_{\max}}{\left(1+e^{\left(\frac{\left(V-V_{50}\right)}{k}\right)}\right)} \end{array}$$

where *G*_*max*_ and *I*_*max*_ are a maximal conductance and maximal current density during the steady-state inactivation, respectively; *V*_50_ is a membrane potential at which 50% of the *G*_*max*_ or *I*_*max*_ values are reached, and *k* is a slope factor. Fitting was done with a MATLAB *fit function* (MathWorks, MA, USA) using a non-linear least-squares method. The best fit was determined by the highest score of the goodness of fit parameter *adjrsquare*. At the end of fitting procedure, the parameter *adjrsquare* was in a range of 0.95–0.99.

The values of ASIC current amplitudes, *I*_*ASIC*_, were fitted with Hill's equation:


(3) $$\begin{array}{l} I_{ASIC}=\frac{I_{\max}}{\left(1+{\left(\frac{H_{50}}{\left[H^{+}\right]}\right)}^{n}\right)} \end{array}$$

to assess the changes, which ASIC channels underwent under CFA-induced inflammation; here *I*_*max*_ refers to the maximal amplitude of *I*_*ASIC*_ evoked in the neurons by a fast application of Tyrode's solution having different pH values, *H*_50_ is [H^+^], at which the ASIC current has an amplitude equal to the half of *I*_*max*_, and *n* is a Hill's coefficient.

The statistical comparisons were performed using unpaired Student's *t*-test and ANOVA test in Origin 8.0 (Microcal Software, MA, USA). Differences between the mean values were considered to be significant when *p* < 0.05. Normality of data distribution was checked using Shapiro–Wilk test in Origin 8.0.

## Results

### Inflammation Causes Increase of Excitability of Caps^–^lpH^+^ DRG Neurons

Taking into account that inflammation is one of the pathogenic complications of long-term diabetes and that caps^−^lpH^+^ DRG neurons demonstrated increased excitability in a model of long-term STZ-induced diabetes (Duzhyy et al., 2015), we expected to observe an increase in excitability of the caps^−^lpH^+^ DRG neurons in a CFA model of peripheral inflammation. Indeed, it appeared that the AP threshold (APT), a parameter directly reflecting the excitability, significantly decreased under the CFA inflammation [−37.7 ± 1.5 mV in control (*n* = 9 from 3 rats) vs. −42.5 ± 1.1 mV under inflammation (*n* = 9 from 3 rats, *p* < 0.03)] (Figures 1B,C). At the same time, the ADP/AHP area was not significantly changed [0.13 ± 0.08 mV^*^s in control (*n* = 9 from 3 rats) vs. 0.17 ± 0.17 mV^*^s under CFA-induced inflammation (*n* = 9 from 3 rats, *p* > 0.8)] (Figure 1D). Thus, while the significant decrease of an AP threshold under CFA-induced inflammation was similar to a decrease under the STZ-induced diabetes (Duzhyy et al., 2015), an ADP/AHP area, reflecting a contribution of T-type channels to AP generation, did not change under the CFA-induced inflammation contrary to its significant increase under the STZ-induced diabetes (Duzhyy et al., 2015). These results demonstrated an increased excitability of the caps^−^lpH^+^ neurons under the CFA-induced inflammation and suggested an altered contribution of T-type channels and/or possible involvement of other channels in an increase of neuronal excitability under the inflammation compared with the STZ-induced diabetes (Duzhyy et al., 2015).

Therefore, we decided to check if the AP overshoot, the other parameter characterizing the neuronal excitability, was changed under CFA-induced inflammation. Its increase under inflammation would suggest upregulation of voltage-gated Na^+^ current. Indeed, inflammation significantly increased the AP overshoot to 42.8 ± 1.7 mV (*n* = 20 from 5 rats) compared with 33.4 ± 3.6 mV in control (*n* = 18 from 5 rats, *p* < 0.05) (Figure 1E).

Thus, under CFA-induced inflammation, the AP threshold decreased and the AP overshoot increased in the caps^−^lpH^+^ DRG neurons indicating an increase in the neuronal excitability.

### Upregulation of Voltage-Gated Na^+^ Channels and Changes in Gating Properties of T-type Ca^2+^ Channels Under CFA-Induced Inflammation

**Figure 2:**
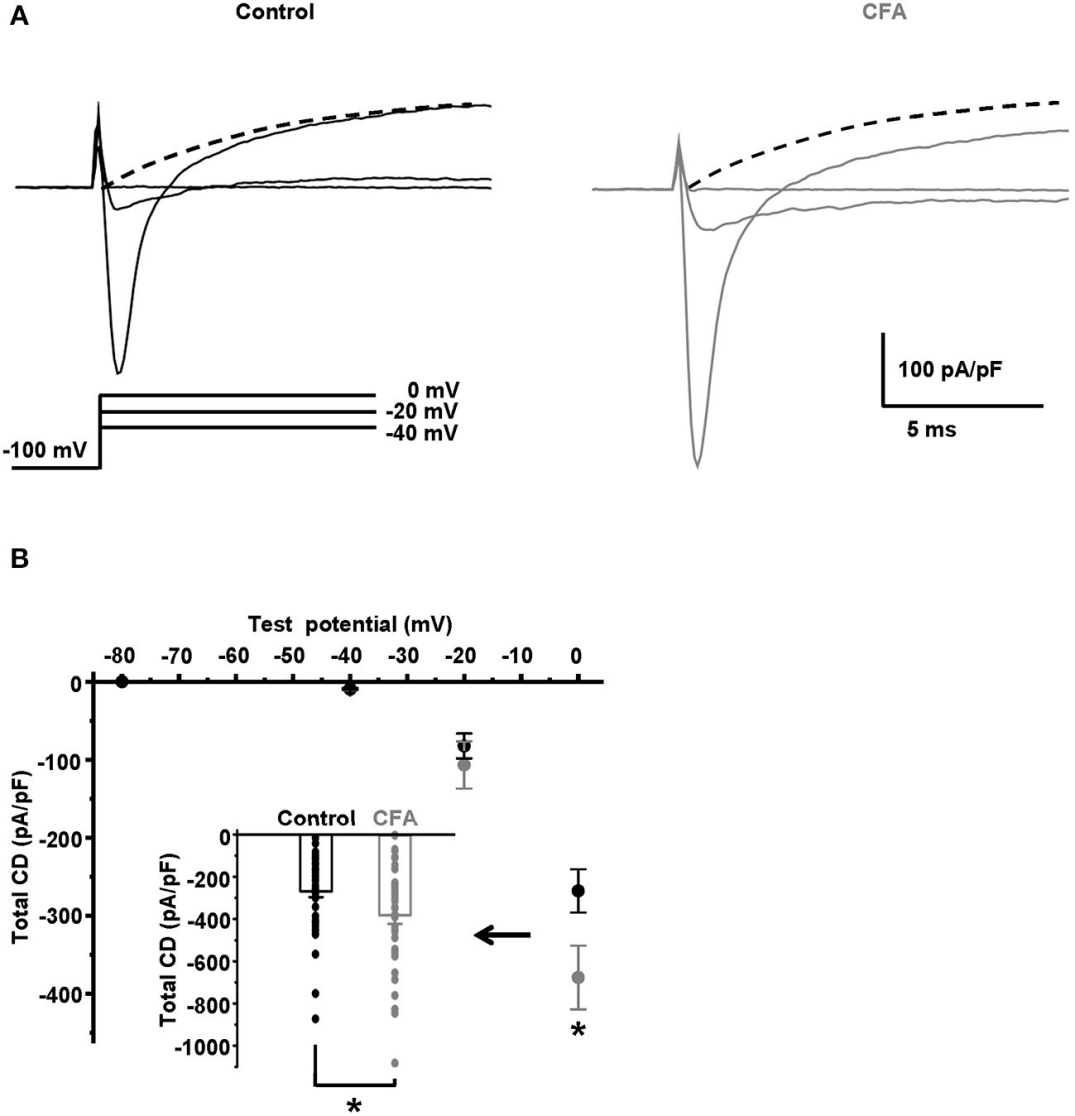
Total voltage-gated current is significantly increased in the caps^−^lpH^+^ neurons under CFA-induced inflammation. **(A)** The representative traces of total voltage-gated currents, such as Na^+^, Ca^2+^, and K^+^ currents, recorded in Tyrode's solution in neurons from the control (black) and inflamed (gray) rats. An activation protocol is shown below the traces. The dashed lines are extrapolations of K^+^ current evoked by a depolarization step to 0 mV to an onset of stimulations. **(B)** A plot of total current density for neurons from the control (black) and inflamed (gray) rats vs. test potential in the range from −80 to 0 mV. An insert shows the current densities at a depolarization step to 0 mV. *p = 0.024.

**Figure 3:**
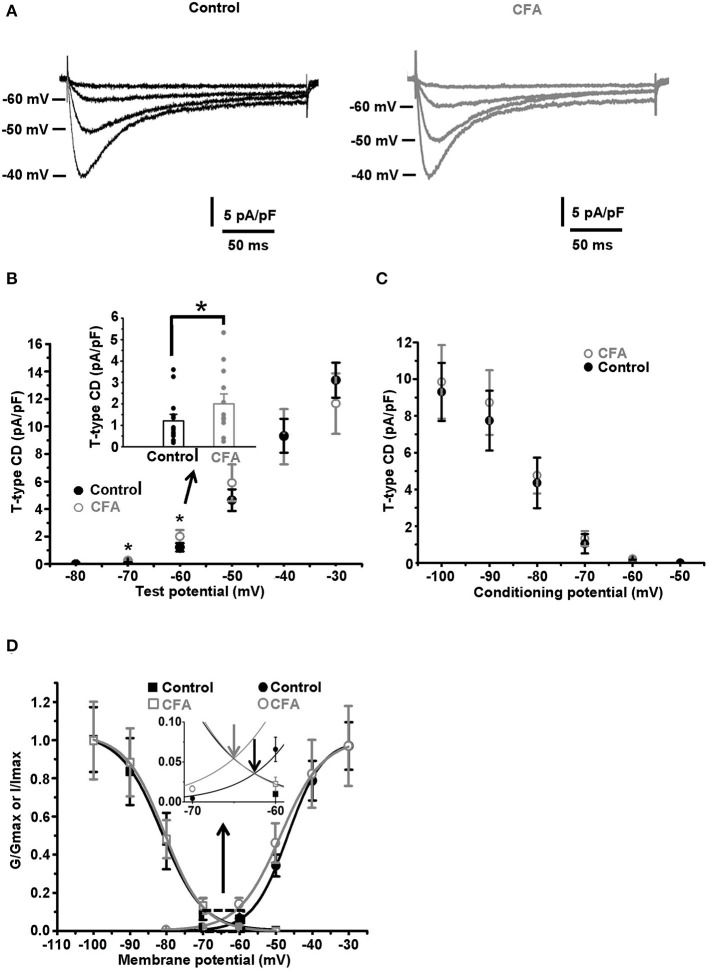
Changes in the gating properties of T-type channels of the caps^−^lpH^+^ neurons under CFA-induced inflammation. **(A)** The averaged current traces demonstrate upregulation of the T-type current under CFA-induced inflammation (*n* = 5 neurons for both control animals, left, and animals with inflammation, right). **(B)** A plot of T-type current density for the caps^−^lpH^+^ neurons from the control (black) and inflamed (gray) rats. The T-type currents were evoked by the depolarization steps from a holding potential of −100 mV (3.5 s) to test potentials from −80 mV through 0 mV (250 ms) with 10 mV increment. A significant increase of T-type current density was revealed at voltage steps to −60 and −50 mV, rather than to −40 mV and above. An insert shows the T-type current densities at a depolarization step to −60 mV. **(C)** A plot of steady state inactivation of T-type current density. The T-type currents were recorded at depolarization step to −40 mV (250 ms) from holding potentials ranging from −100 to −50 mV (3.5 s) with 10 mV increment. No significant changes in the steady-state inactivation of T-type current were observed under CFA-induced inflammation. **(D)** Fitting of steady-state activation and inactivation with Boltzmann function reveals an increase in the normalized conductance of T-type channels (right curves) under CFA-induced inflammation. An insert demonstrates that the inflammation increased a non-inactivating “window” T-type current. Arrows show that a peak of the “window” current was substantially increased. ^*^p < 0.05.

Because of the significant increase in the AP overshoot, we hypothesized that a voltage-gated Na^+^ current should be increased in the caps^−^lpH^+^ DRG neurons under CFA-induced inflammation. By estimations described below, the Na^+^ current comprised more than 90% of the total voltage-gated current at its peak at a depolarization step from −100 to 0 mV. The total current density was more than 250 pA/pF (Figure 2B), while the corresponding value for T-type current was estimated at about 20 pA/pF for T-type current (Figure 3B) or even less than that for K^+^ current (dashed lines in Figure 2A). Besides, T-type Ca^2+^ and K^+^ currents were oppositely directed and should almost cancel each other. Thus, the amplitude of Na^+^ current at a depolarization step to 0 mV was estimated as the amplitude of the total voltage-gated current in both the control and inflammatory conditions. Using these estimations, we found that under inflammation, the voltage-gated Na^+^ current density increased by 40% at a depolarization step from −100 to 0 mV [from 268.3 ± 27.8 pA/pF in control neurons (*n* = 42 from 8 rats) to 379.4 ± 40.8 pA/pF in neurons of inflamed rats (*n* = 37 from 7 rats, *p* = 0.024)] (Figure 2).

Fitting a total membrane conductance with the Boltzmann function [as shown in Equation (1)] demonstrated a significant increase of maximal conductance, *G*_*max*_, under inflammation. *G*_*max*_ increased from 5.8 ± 0.5 pA/(pF^*^mV) in control (*n* = 42 from 8 rats) to 8.2 ± 0.8 pA/(pF^*^mV) under inflammation (*n* = 37 from 7 rats, *p* = 0.014). At the same time, the membrane potential of half activation, *V*_50_, did not change significantly [−8.2 ± 1.2 mV in control (*n* = 42 from 8 rats) and −6.9 ± 1.6 mV under inflammation (*n* = 37 from 7 rats, *p* > 0.5)]; nor did the slope factor *k* [5.6 ± 0.2 mV in control (*n* = 42 from 8 rats) and 5.3 ± 0.2 mV under inflammation (*n* = 37 from 7 rats, *p* > 0.3)]. Taking into account that CFA-induced inflammation did not significantly change K^+^ and Ca^2+^ currents in the range of voltage steps between −40 and 0 mV (data not shown), we concluded that *G*_*max*_ of voltage-gated Na^+^ current was increased under inflammation while *V*_50_ and *k* were not significantly changed. The increased *G*_*max*_ should contribute to a decreased AP threshold observed in the caps^−^lpH^+^ neurons under CFA-induced inflammation.

It has been previously established that a low AP threshold of the caps^−^lpH^+^ DRG neurons (below −40 mV) is mainly due to T-type current activation (Duzhyy et al., 2015). Thus, we hypothesized that the changes in the functioning of T-type Ca^2+^ channels, along with the increased sodium conductance, may underlie the decrease of the AP threshold under CFA-induced inflammation. So, we looked at the T-type current changes under inflammation. As appeared, the T-type current density significantly increased in the neurons of rats with inflammation at the depolarization steps from −100 to −70 mV and to −60 mV, while no significant changes were observed at the depolarization steps to −50 and −40 mV, respectively (Figures 3A,B). For example, the current density at a depolarization step to −60 mV was increased by 64% [2.0 ± 0.4 pA/pF under inflammation (*n* = 12 from 3 rats) vs. 1.2 ± 0.2 pA/pF in control (*n* = 13 from 3 rats, *p* < 0.05)], while no changes were observed at depolarization to −40 mV [9.3 ± 2.0 pA/pF under inflammation (*n* = 12 from 3 rats) vs. 9.3 ± 1.2 pA/pF in control (*n* = 13 from 3 rats, *p* > 0.97)] (Figure 3B). Fitting these data with the Boltzmann function is shown in Figure 3D and demonstrates significant changes in the membrane potential of half activation, *V*_50_, and slope factor, *k*, observed under CFA-induced inflammation. In particular, in neurons of inflamed rats, *V*_50_ was shifted by about 2 mV in the hyperpolarizing direction [from −46.8 ± 0.6 mV in control (*n* = 13 from 3 rats) to −48.5 ± 0.4 mV under inflammation (*n* = 12 from 3 rats, *p* = 0.03)], while *k* increased by 1 mV [from 4.8 ± 0.2 mV in control (*n* = 13 from 3 rats) to 5.8 ± 0.3 mV under inflammation (*n* = 12 from 3 rats, *p* = 0.006)] (Figure 3D). At the same time, the maximal conductance *Gmax* of the Boltzmann function fit was not significantly changed under inflammation [0.16 ± 0.03 pA/(pF^*^mV), *n* = 12 from 3 rats] compared with control [0.17 ± 0.02 pA/(pF^*^mV), *n* = 13 from 3 rats, *p* > 0.8] (Figure 3D).

Contrary to the activation parameters, no significant changes for the parameters of steady-state inactivation were found under inflammation. *I*_*max*_ was 10.0 ± 2.0 pA/pF and 9.8 ± 1.6 pA/pF, *p* > 0.5; *k* was 4.4 ± 0.2 and 4.9 ± 0.7, *p* > 0.3; *V*_50_ was −79.9 ± 1.4 mV and −82.1 ± 2.3 mV, *p* > 0.4, under inflammation (*n* = 14 from 3 rats) and in control (*n* = 5 from 3 rats), correspondingly (Figures 3C,D).

A range of membrane potentials, in which the T-type current activation and inactivation curves are overlapped, is the one where a non-inactivating (window) current occurs. This current contributes to the oscillations of resting membrane potential (MPOs) and spontaneous firing of the neurons (Hughes et al., 1999; Crunelli et al., 2005; Chevalier et al., 2006, 2008; Amarillo et al., 2014). In this study, a significant shift of the activation curve of T-type current in a hyperpolarizing direction under inflammation led to a significant increase in a peak of T-type “window” current, by about 67%, and its shift in the hyperpolarizing direction by 2.7 mV (an insert in the Figure 3D, *n* = 12 from 3 rats under inflammation and *n* = 13 from 3 rats in control, *p* < 0.05).

There was no significant change in a density of K^+^ current under CFA-induced inflammation using an activation protocol of membrane depolarization from a holding potential of −100 to −60 mV through +40 mV with a 20-mV increment (data not shown).

Thus, in caps^−^lpH^+^ DRG neurons, CFA-induced inflammation resulted in a functional upregulation of Na^+^ channels and changes in the gating properties of T-type channels, which both contribute to a decrease in the AP threshold.

### Sensitization of Caps^–^lpH^+^ DRG Neurons to Protons Under CFA-Induced Inflammation

**Figure 4:**
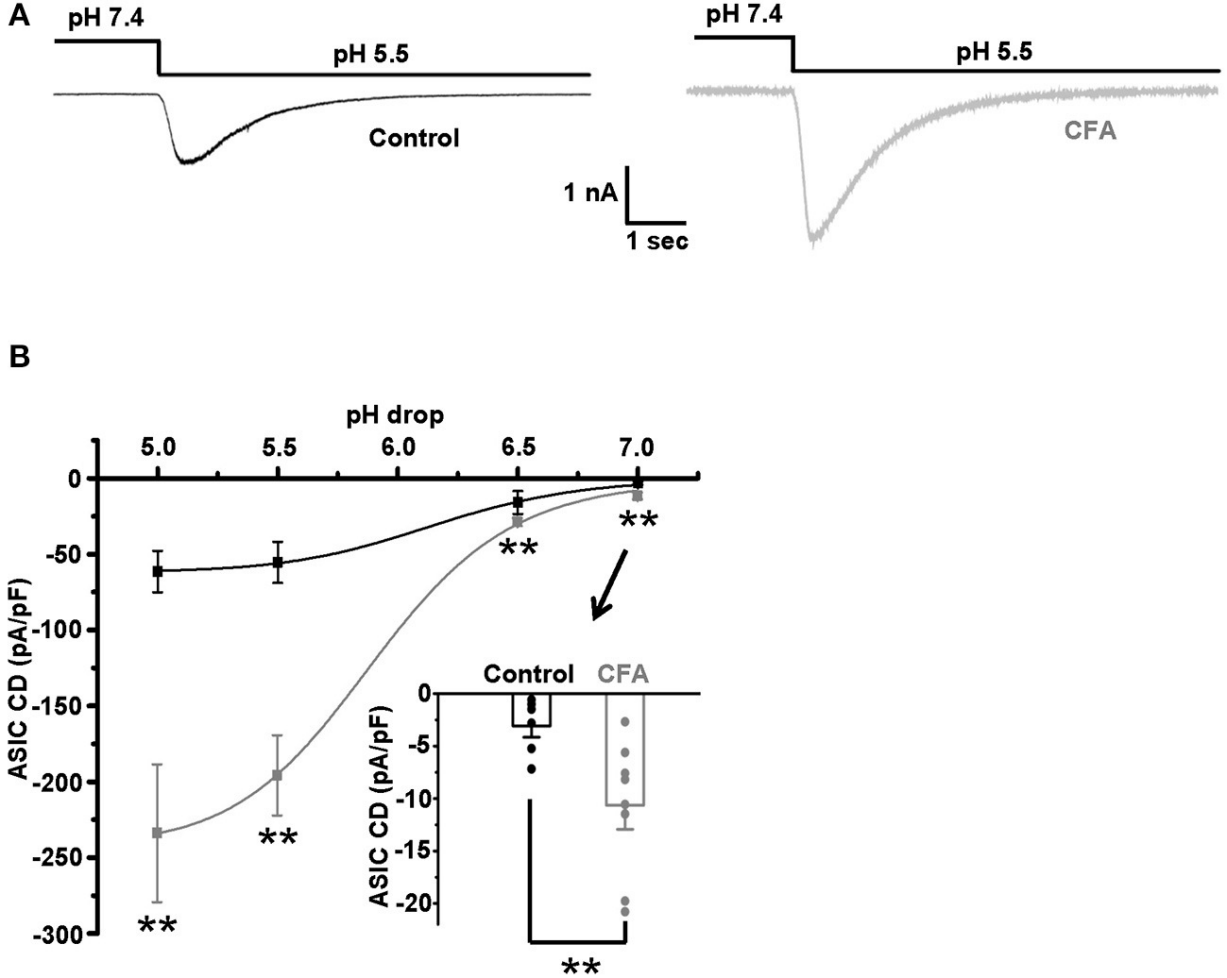
The upregulation of acid-sensing ion channels (ASICs) in the caps^−^lpH^+^ DRG neurons under CFA-induced inflammation. **(A)** The representative traces demonstrate an increase of ASIC-mediated current in the caps^−^lpH^+^ neurons under CFA-induced inflammation. **(B)** Pooled results showing a significant increase of amplitudes of ASIC-mediated current under inflammation. The currents were evoked by a sharp decrease of pH of extracellular solution. The curves are Hill's equation fits of the datasets. An insert demonstrates that the inflammation increased ASIC-mediated current at pH drop to 7.0. ^**^p < 0.02.

The sensitivity to protons, established for the caps^−^lpH^+^ DRG neurons as an ability of their plasma membrane to conduct an inward current in a voltage-clamp mode or generate an AP in a current-clamp mode in response to a decrease of extracellular pH, is conferred to these neurons by ASICs (Duzhyy et al., 2015). So, sensitization of the plasma membrane to protons, in other words, an increase of sensitivity to protons, should be expressed as an increase of ASICs-mediated current in a voltage-clamp mode and a decrease of a threshold pH drop from a physiological pH 7.4 evoking an AP generation in a current-clamp mode. It has been earlier established that the expression of ASIC mRNAs is increased in small-sized nociceptors under inflammation (Voilley et al., 2001). Thus, we expected to detect an upregulation of ASICs-mediated current in the caps^−^lpH^+^ DRG neurons under the CFA-induced inflammation. Indeed, the inflammation led to a 3-fold increase of the ASIC current density in response to the applications of low-pH external solutions, having pH in a range of 5.0–7.0 (Figure 4). In particular, the current density was increased from 55.4 ± 13.6 pA/pF in control to 194.7 ± 26.5 pA/pF under inflammation at pH drop from 7.4 to 5.0 and from 3.1 ± 1.1 pA/pF in control to 10.7 ± 2.3 pA/pF under inflammation at pH drop from 7.4 to 7.0 (Figure 4B, *n* = 6 from 3 rats in control and *n* = 8 from 3 rats under inflammation, *p* < 0.02). The fitting plots for ASIC peak current density vs. pH with Hill's function (Figure 4B) showed a significant difference between the inflammatory and control conditions for the maximal amplitude of current density, *I*_*max*_ [−62.7 ± 2.0 pA/pF in control (*n* = 6 from 3 rats) and −244.5 ± 8.0 pA/pF under inflammation (*n* = 8 from 3 rats, *p* < 0.05)], but not for pH of half activation, *pH*_50_ [6.17 ± 0.06 in control (*n* = 8 from 3 rats) and 5.90 ± 0.04 under inflammation (*n* = 8 from 3 rats, *p* > 0.05)], or Hill's coefficient *n* [1.39 ± 0.12 in control (*n* = 8 from 3 rats) and 1.45 ± 0.12 under inflammation (*n* = 8 from 3 rats, *p* > 0.05)]. Thus, the transient component of ASIC current was upregulated under CFA-induced inflammation without the changes in its pH-dependence.

A persistent component of ASIC current, if present, may also influence the excitability of DRG neurons (Yagi et al., 2006). Therefore, the existence of this current in the caps^−^lpH^+^ DRG neurons and its possible changes under inflammation were tested. In control neurons, the persistent current was negligible at pH 7.0 and 6.5, while at pH 5.5 its density was −0.43 ± 0.05 pA/pF (*n* = 6 from 3 rats). The persistent current at pH 5.5 was increased under inflammation, about 3-fold (from −0.43 ± 0.05 pA/pF in control, *n* = 6 from 3 rats, to −1.5 ± 0.3 pA/pF under inflammation, *n* = 8 from 3 rats, *p* < 0.05) and became measurable at pH 6.5 (−0.40 ± 0.04 pA/pF, *n* = 8 from 3 rats).

**Figure 5:**
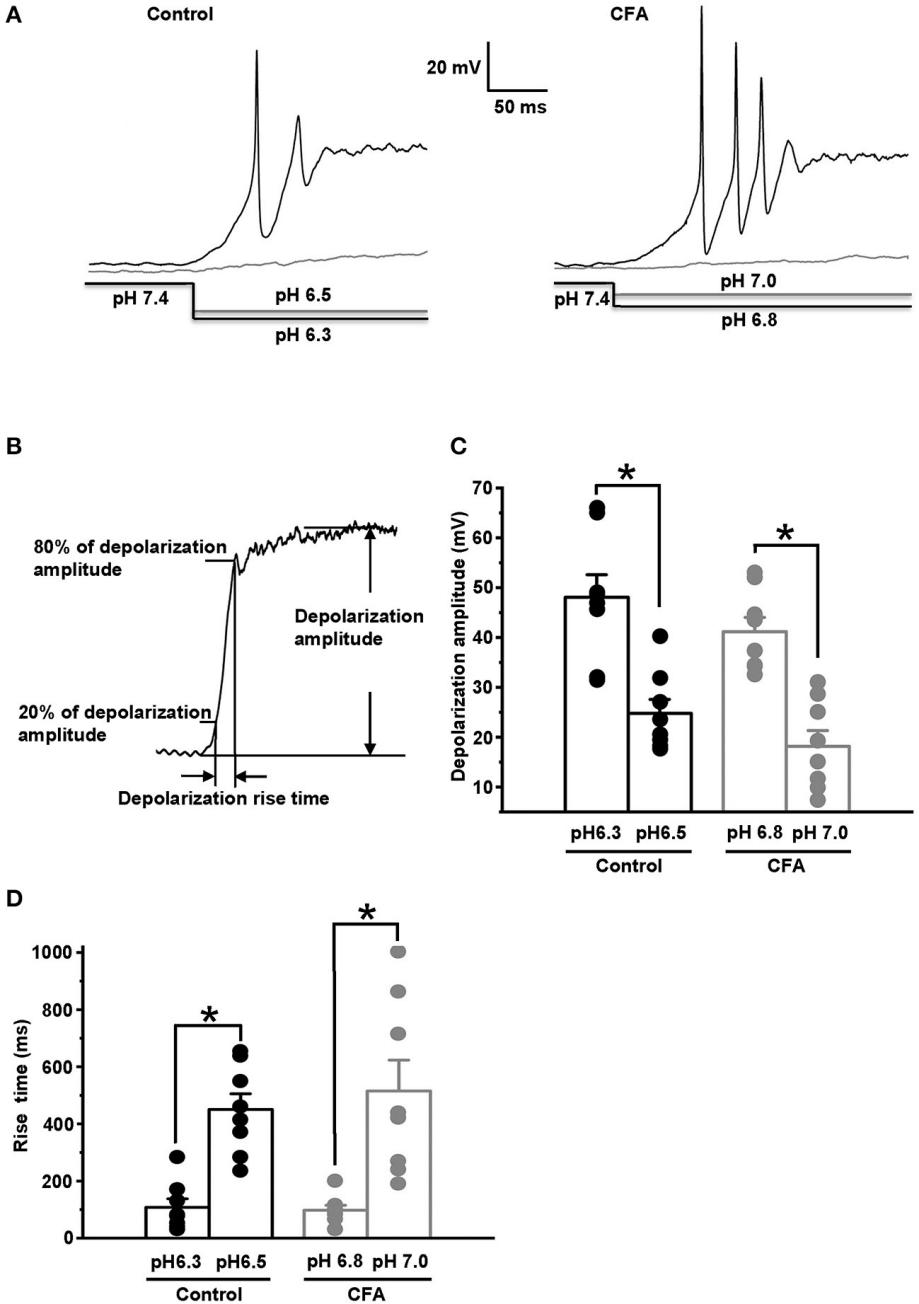
Complete Freund's adjuvant-induced inflammation shifts pH threshold for AP generation in the caps^−^lpH^+^ neurons. **(A)** The representative traces showing APs generated in the caps^−^lpH^+^ neurons of control (left) and inflamed (right) rats at pH drop in the extracellular solution from a physiological to threshold level. About 25% of the neurons generated AP discharges in a response to the threshold pH drop. At slightly smaller pH drops (by 0.2 units), the depolarization was significantly slower and smaller. The pH changes in the external solutions are shown below the traces. **(B)** The parameters of depolarization potential evoked by pH drop: its amplitude and rise time. **(C)** The amplitudes of depolarization evoked by pH drops, which were slightly (by 0.2 units) above the thresholds, were significantly smaller compared with the ones evoked by threshold pH drops in the caps^−^lpH^+^ neurons of control and inflamed rats. The mean values are presented as bars, single values—as circles. **(D)** The rise time of depolarization evoked by the subthreshold (by 0.2 units) pH drop was significantly longer than the ones evoked by threshold pH drops in the caps^−^lpH^+^ neurons of the control and inflamed rats. ^*^p < 0.05.

We suggested that the upregulation of ASIC current under CFA-induced inflammation could produce a decrease in a threshold for pH drop evoking an AP generation. The value of this threshold was estimated by stepwise pH drops from physiological pH value of 7.4 while holding the neuron membrane potential at −60 mV in a current clamp mode. About 25% of the neurons in the control and inflammatory groups generated APs in response to drops to 6.3 and 6.8, respectively (Figure 5A left and right, *n* = 8 from 3 rats for each group). At slightly smaller pH drops of external solutions applied to the same groups of neurons (to pH 6.5 for the control group and 7.0 for CFA group), AP generation was not observed (Figure 5A left and right). Thus, pH 6.3 for the control neurons and pH 6.8 for the neurons under inflammation were considered as 25% pH threshold levels for AP generation (pH_0.25_ thresholds). In the caps^−^lpH^+^ neurons of the control and CFA groups, pH drops to pH_0.25_ thresholds resulted in a fast and strong depolarization (Figures 5B–D). At slightly smaller pH drops (by 0.2 units), the depolarization was significantly slower and smaller (Figures 5B–D). In particular, the depolarization amplitude in the control neurons was 24.8 ± 2.8 mV (at pH 6.5) compared with 48.1 ± 4.5 mV at the threshold pH of 6.3 (*n* = 8 from 3 rats, *p* < 0.05). In the neurons of inflamed rats, it was 18.4 ± 3.2 mV at pH 7.0 compared with 41.4 ± 2.9 mV at the threshold pH of 6.8 (*n* = 8 from 3 rats, *p* < 0.05) (Figure 5C). The rise times of depolarization were increased in the control neurons from 108 ± 30 ms at the threshold pH of 6.3 to 451 ± 55 ms at pH of 6.5 [*n* = 8 from 3 rats (*p* < 0.05)] and in the neurons from inflamed rats, from 97 ± 17 ms at the threshold pH of 6.8 to 515 ± 108 ms at pH 7.0 [*n* = 8 from 3 rats (*p* < 0.05)] (Figure 5D). It is interesting to note that the APs were not generated in the neurons at the subthreshold pH drops although the membrane potentials (−35.2 mV for the control neurons and −41.6 mV for the neurons from inflamed rats) reached thresholds for AP generation evoked by the current injections (Figure 1C). Most probably, it might be explained by slow rises of the depolarization potential evoked by the subthreshold pH drops that resulted in the inactivation of voltage-gated channels contributing to AP generation.

Thus, under CFA-induced inflammation, the expression and/or conductance of ASICs was significantly increased without changes in their activation properties. It resulted in a decrease of a threshold pH drop evoking an AP generation. The persistent ASIC current also increased under inflammation, although at physiologically relevant pH its value was too low to induce AP generation.

### Acidification Increases the AP Threshold for the Caps^–^lpH^+^ DRG Neurons Due to Proton-Dependent Modulation of T-type Channels

**Figure 6:**
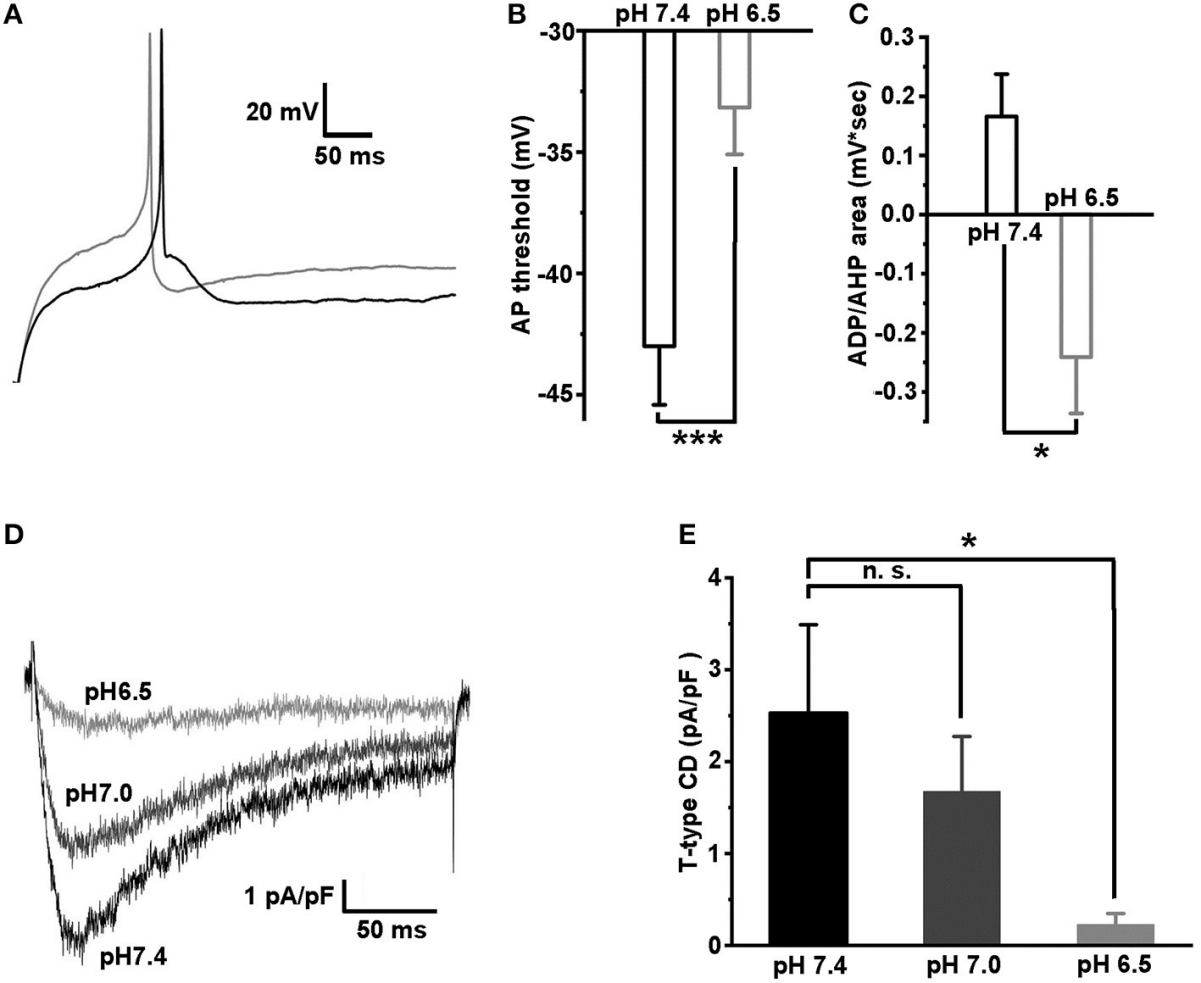
Acidification of extracellular solution downregulates the excitability of caps^−^lpH^+^ neurons due to inhibition of T-type current. **(A)** The representative traces showing an increase in the AP threshold and conversion of ADP to AHP after a change of pH in the extracellular solution from 7.4 (black trace) to 6.5 (gray trace). The APs were evoked by the threshold current injections. **(B,C)** The pooled results demonstrate a significant increase of the AP threshold and conversion of ADP to AHP, when pH of the extracellular solution was changed from 7.4 to 6.5. **(D,E)** Inhibition of T-type current *via* acidification of extracellular solution. The representative traces of T-type current for the same cell evoked by a depolarization step from −100 to −50 mV at pH of the extracellular solution 7.4 (black trace), 7.0 (dark gray trace), and 6.5 (gray trace). The pooled results demonstrating significant inhibition of T-type current at pH 6.5. ^***^p < 0.001 and ^*^p < 0.05.

Previously, it was established that the inflammation causes a local acidosis at a site of inflammation (Edlow and Sheldon, 1971; Simmen et al., 1994; Naghavi et al., 2002; Schomack and Gillies, 2003). We hypothesized that the excitability of caps^−^lpH^+^ neurons would change under extracellular acidosis because T-type channels activation properties were found to depend on the pH of extracellular solution (Delisle and Satin, 2000; Talavera et al., 2003; Park et al., 2013). So, we checked if a decrease of pH of extracellular solution changed excitability of the caps^−^lpH^+^ DRG neurons under normal conditions (Figure 6). In solutions having pH in a range from 5.5 to 6.5, a significant increase of the AP threshold was observed compared with pH 7.4 (Figures 6A,B). It is interesting to note that the increase was accompanied by the conversion of ADP to AHP (Figures 6A,C). For example, the AP threshold increased from −43.0 ± 2.4 mV at pH 7.4 to −33.2 ± 1.9 mV at pH 6.5 (Figure 6B, *n* = 8 from 3 rats, *p* < 0.001) that was complemented by a decrease of the ADP/AHP area from 0.17 ± 0.07 mV^*^s to −0.24 ± 0.10 mV^*^s (Figure 6C, *n* = 8 from 3 rats, *p* = 0.04). A similar increase in the AP threshold and related decrease of ADP/AHP area were obtained for the caps^−^lpH^+^ DRG neurons when the T-type current was inhibited by Ni^2+^ and amiloride (Duzhyy et al., 2015). Therefore, we further checked if T-type current could be substantially inhibited at pH of external solution of 6.5 compared with the pH 7.4 to account for the conversion of ADP to AHP. We found that at pH 6.5, the T-type current was almost completely inhibited, by 94 ± 2%, at a depolarization step from −100 to −50 mV (from 2.53 ± 0.96 pA/pF at pH 7.4 to 0.23 ± 0.12 pA/pF at pH 6.5, *n* = 6 from 3 rats, *p* < 0.05) (Figures 6D,E). The T-type current had also tendency for proton-induced inhibition at pH 7.0 resulting in a non-significant decrease of the amplitude, by 33 ± 6%, at the same depolarization step (from 2.53 ± 0.96 pA/pF at pH 7.4 to 1.67 ± 0.60 pA/pF at pH 7.0, *n* = 6 from 3 rats, *p* = 0.068) (Figures 6D,E).

Thus, the T-type channels in the caps^−^lpH^+^ DRG neurons are inhibited by the acidification of extracellular solution that results in a related decrease of neuronal excitability.

### Spontaneous Activity of Caps^–^lpH^+^ DRG Neurons Is Increased Under CFA-Induced Inflammation

**Figure 7:**
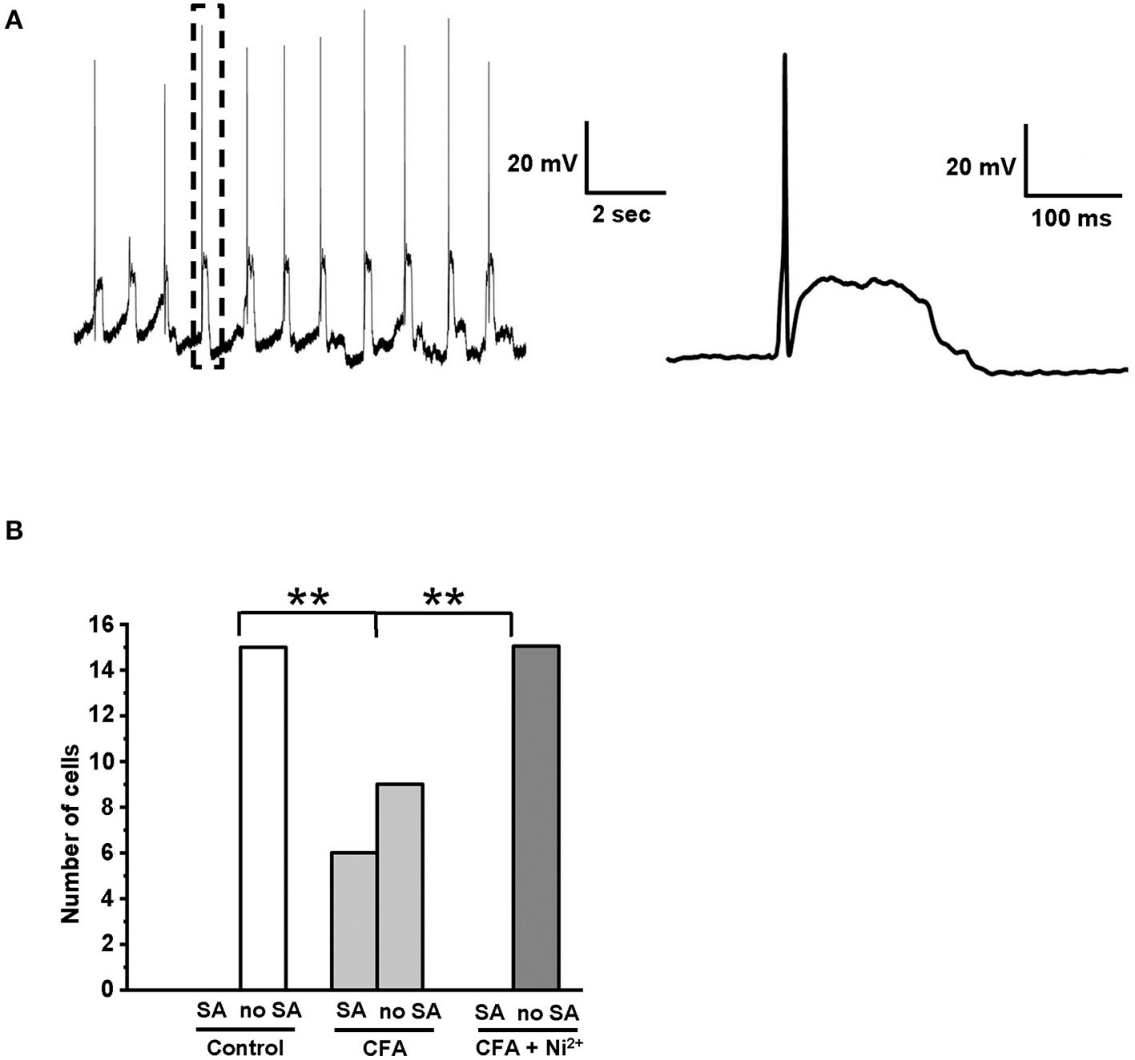
Spontaneous AP generation was developed in the caps^−^lpH^+^ neurons under CFA-induced inflammation. **(A)** A representative trace shows an example of spontaneous activity observed in the caps^−^lpH^+^ neuron obtained from a rat with CFA-induced inflammation. The activity represents regularly emerging APs followed by a phase of prolonged stationary depolarization. A single AP followed by a phase of prolonged stationary depolarization enclosed in a box is shown to the right with a higher time resolution. **(B)** A plot representing a proportion of caps^−^lpH^+^ neurons having spontaneous activity (SA). The neurons were isolated from the control rats or rats with CFA-induced inflammation. In the latter case, the neurons were tested with or without Ni^2+^ (T-type channel blocker) in the extracellular solution. In this study, 15 neurons were recorded in each group. ^**^p < 0.02.

Previously, Chevalier and colleagues established a pivoting role of “window” T-type current in a specific type of membrane potential oscillations (MPOs) consisting of regularly emerging APs followed by a phase of prolonged stationary depolarization (Chevalier et al., 2006, 2008, 2012). We hypothesized that an observed increase of the “window” T-type current in the caps^−^lpH^+^ neurons under inflammation (Figure 3D) could lead to the development of MPOs. Indeed, 6 out of 15 caps^−^lpH^+^ neurons from the four animals with inflammation showed this type of activity (Figure 7A), while no neurons from the control or inflamed animals with 50 μM Ni^2+^ added to the bath solution did (Figure 7B; 15 cells from 4 rats in each group, *p* < 0.02; Fisher's exact test). Thus, the development of MPOs and spontaneous firing in the caps^−^lpH^+^ neurons is associated with an increase of “window” T-type current under inflammation. Besides, Hughes and coauthors (Hughes et al., 1999) found that a prolonged depolarization phase of MPOs may emerge due to an increase in the T-type current or decrease in the leak current; the latter was not changed in our study also supporting a role for T-type current in MPOs (data not shown). In summary, the MPOs consisting of regularly emerging APs followed by a phase of prolonged stationary depolarization, take place in the caps^−^lpH^+^ DRG neurons of inflamed rats that might contribute to the development of central sensitization and chronic inflammatory pain.

## Discussion

The main finding of this study is that the CFA-induced inflammation leads to the upregulation of Na^+^ and T-type Ca^2+^ voltage-gated ion channels and ASICs in the caps^−^lpH^+^ nociceptive DRG neurons. This upregulation contributes to increased excitability and spontaneous firing of the neurons and may underlie hyperalgesia and chronic inflammatory pain.

### Biophysical Mechanisms of Increased Excitability of Caps^–^lpH^+^ Nociceptors

In this study, the caps^−^lpH^+^ DRG neurons have demonstrated an increased excitability under CFA-induced inflammation. In particular, it was expressed as a decrease of a threshold for AP generation and an increase in AP overshoot in response to the current injections. The modulation of several types of channels under inflammation underlie these changes in AP generation. First, it is an increase of maximal conductance of voltage-gated Na^+^ channels, *G*_*max*_, that definitely contributes to the increase in the AP overshoot. The increase of *G*_*max*_ by 40% without any change in the activation properties of the Na^+^ channels assumes an increased functional expression of the channels. Second, it is the upregulation of T-type current at the membrane potentials below −40 mV that might decrease the AP threshold below −40 mV under inflammation. This T-type current upregulation is due to the modulation of gating properties of T-type channels without changes in their functional expression. In addition to an increase of a slope factor *k*, that increased the window T-type current, we observed a shift of the activation curve in a hyperpolarized direction by about 2 mV (Figure 3D). Similar shift by 2 mV was previously shown to result in a significant increase of neuronal burst-generating capabilities (Tscherter et al., 2011).

The amplitudes of both Na^+^ and T-type Ca^2+^ currents in the caps^−^lpH^+^ neurons are comparable at a depolarization step to −40 mV (Duzhyy et al., 2015) as well as the values of CFA-induced upregulation of these currents as shown in this work. Thus, the inflammatory-induced upregulation of both currents should contribute to an observed decrease of AP threshold. At the same time, the upregulation of Na^+^ current in a range of membrane potentials above −40 mV should account for an increase in the AP overshoot under inflammation. This increase of AP overshoot might lead to an enhanced release of neurotransmitters in the central terminals of caps^−^lpH^+^ neurons contributing to an increased excitation of secondary nociceptors in the spinal cord.

The increased excitability of caps^−^lpH^+^ nociceptors under CFA-induced inflammation was also expressed as a reduction of pH drop evoking AP generation. It has been previously shown that the acidification of skin causes generation of APs in peripheral afferents of primary nociceptors (Steen and Reeh, 1992). Additionally, it has been shown that under inflammation, ASICs undergo upregulation in nociceptive DRG neurons that causes an increase in the percentage of cells generating APs at the same pH drop as in control (Mamet et al., 2002). In this study, we have confirmed that such inflammation-induced upregulation of ASICs takes place in a particular subtype of small-sized nociceptors and is manifested as an increased ASIC-mediated current. Based upon IB4^−^caps^−^lpH^+^ phenotype, small size and current signature, the caps^−^lpH^+^ DRG neurons are similar to Type 3 DRG neurons described by Petruska and colleagues and further studied by Jiang and colleagues (Petruska et al., 2000; Jiang et al., 2006) (although some of the AP properties of Type 3 neurons are not similar to the ones of caps^−^lpH^+^ neurons). Additionally, inactivation for ASIC-mediated current shown for the caps^−^lpH^+^ neurons is very similar to that for Type 3 DRG described by Jiang and colleagues (Jiang et al., 2006). Thus, it is most probably that we studied an inflammatory-induced upregulation of ASICs in the Type 3 DRG neurons. This upregulation may be caused by an increased expression of ASIC mRNA induced by the proinflammatory mediators (Mamet et al., 2002) and by the potentiation of channel conductance by inflammatory stimuli (histamine, 5-HT, arachidonic acid, spermine, FMRFamides, etc.) (Deval and Lingueglia, 2015; Rash, 2017). Thus, the neurons become more sensitized to the protons that leads to a substantial (0.5 unit) decrease of a threshold pH drop evoking AP generation. In particular, it means that under inflammation, a small drop of pH from a physiological level of 7.4 to 6.8 is sufficient to generate APs in this type of nociceptive neurons. Considering that the frequent degranulation of mast cells under inflammation leads to the pH fluctuations below the physiological level (Williams and Webb, 2000), the observed sensitization of caps^−^lpH^+^ nociceptors to protons may result in their frequent firing and concomitant pain sensation.

The ASIC1a, ASIC1b, and ASIC3 subunits are highly expressed in the peripheral sensory neurons (Deval and Lingueglia, 2015) with a specific expression of ASIC1b in the peripheral nervous system (PNS) (Chen et al., 1998). The pharmacological (Yu et al., 2010) and genetic (Deval et al., 2011; Walder et al., 2011) experiments strongly argue for the involvement of ASIC3 channels in the peripheral nociception and specifically in CFA-induced primary hyperalgesia (Karczewski et al., 2010). Recent data have also emphasized the role of peripheral ASIC1 (both ASIC1a and ASIC1b) in nociception (Deval and Lingueglia, 2015). The ASICs expressed in the nociceptive caps^−^lpH^+^ neurons revealed the time of inactivation in a range of 0.6–1.2 s and *pH*_50_ about 6.0 implying that ASIC1b (having the inactivation time 1–2 s and *pH*_50_ 5.9–6.3 [Rash, 2017]) strongly contribute to the ASIC current in these neurons. At the same time, in the caps^−^lpH^+^ neurons faster inactivation than in ASIC1b and a persistent ASIC current are observed suggesting of the expression in these neurons of ASIC3-mediated component with the inactivation time 0.3 s (Rash, 2017). Notably, the ASIC1a channels have *pH*_50_ of 6.4–6.7 (Rash, 2017). It has been shown that the cleavage of ASIC1a heterologously expressed in oocytes by trypsin results in the change of *pH*_50_ to 6.0 (Vukicevic et al., 2006). However, such cleavage is unlikely in our experiments, since an experimental procedure of DRG neuron isolation by a mixture of trypsin and collagenase similar to ours produced neurons with intact ASIC1a (Poirot et al., 2006). Thus, it is most probably that the cap^−^lpH^+^ nociceptive neurons express a mixture of ASIC1b and ASIC3 channels.

In this work, we have found that the T-type channels of the caps^−^lpH^+^ neurons are strongly inhibited by acidification as previously established for the T-type channels in heterologous systems (Delisle and Satin, 2000; Talavera et al., 2003; Park et al., 2013). In normal conditions when pH threshold level for AP generation is 6.3, the T-type channels are almost completely inhibited and cannot contribute to the AP generation. Inflammation-induced sensitization of these neurons to protons shifts a pH threshold for AP generation to more physiological values (pH 7.0), at which the T-type channels are only slightly inhibited and may substantially contribute to AP generation. This suggestion is based on an inhibitory effect of low pH on T-type channels (Figure 6). Under inflammation, the T-type channels most likely undergo modifications (Cai et al., 2021), so the effect of mild acidification (pH 7.0) on T-type channels and AP threshold may be different and should be additionally studied. Thus, our results demonstrate that an interplay between the ASICs and T-type channels may contribute to an enhanced AP generation of the caps^−^lpH^+^ nociceptors under the inflammatory conditions.

### Potential Role of T-type “Window” Current in Increased Spontaneous Firing of Caps^–^lpH^+^ Neurons Under Inflammation

In this study, a significant increase in spontaneous firing of caps^−^lpH^+^ DRG neurons under inflammation has been observed. Particularly, one type of spontaneous activity, MPOs with a phase of prolonged stationary depolarization, were found at a significantly higher rate under inflammation. This type of MPOs emerges in the neurons expressing T-type channels if the “window” T-type current exceeds the leak current (Hughes et al., 1999). The MPO generation can be driven solely by the T-type current (Chevalier et al., 2006), although other currents, such as I_h_ and I_CAN_, were found to modulate the kinetic parameters of MPOs (Crunelli et al., 2005). Since the leak current was not significantly changed in the caps^−^lpH^+^ neurons under inflammation, and the spontaneous activity was not observed in the neurons from inflamed rats when the T-type channels were blocked, we consider that the increased MPO rate was due to an inflammatory-induced increase of T-type “window” current.

Thus, under CFA-induced inflammation, the caps^−^lpH^+^ nociceptors demonstrate spontaneous activity presumably caused by the upregulation of T-type channels at a subthreshold level. This activity increases spontaneous firing of the neurons and may contribute to the chronic pain sensations under inflammatory conditions.

### Changes of T-type Channels of Caps^–^lpH^+^ Neurons Under STZ-Induced Diabetes and CFA-Induced Inflammation

In this work, we have studied the modulation of T-type channels of caps^−^lpH^+^ neurons in a model of CFA-induced inflammation while previously the same was done for a model of STZ-induced diabetes (Duzhyy et al., 2015). Both models include inflammation component (Shanmugam et al., 2003; Stills, 2005), although inflammation under STZ-induced diabetes develops systemically, while in a CFA-induced model it is localized in a foot of a hind paw. This difference may partially explain distinctions in the T-type channel modulation shown in our works. What seems more important is that the model of CFA-induced inflammation does not include hyperglycemia, the factor that causes changes in the expression of T-type channels under diabetic conditions (Lazniewska et al., 2016). It appears that under CFA-induced inflammation, the maximal conductance of T-type channels is not changed in the caps^−^lpH^+^ neurons while it is increased under STZ-induced diabetes (Duzhyy et al., 2015). Since the maximal conductance is dependent on the density of functionally active channels, its increase in the diabetic conditions is likely due to hyperglycemia-driven enhancement of trafficking of T-type channels to the plasma membrane observed in diabetes (Lazniewska et al., 2016).

The gating properties of T-type channels of the caps^−^lpH^+^ neurons are significantly modified under inflammation while they are not changed in STZ-diabetes. An increase in a slope factor *k* for the T-type current activation could stem from redox modifications of the T-type channels (Todorovic et al., 2001; Nelson et al., 2005, 2007; Joksovic et al., 2006; Orestes et al., 2011; Lee et al., 2013) due to the changes in a redox state of inflamed tissue (Singh and Vinayak, 2015). A shift of T-type current activation curve could be a result of T-type channel phosphorylation *via* the CaMKII pathway (Wolfe et al., 2002; Welsby et al., 2003). This pathway may be activated due to an increased activity of ASIC-expressing neurons under inflammation (Wan et al., 2019).

We have concluded that the upregulation of T-type channels in the caps^−^lpH^+^ nociceptors observed in STZ-induced diabetes is likely due to hyperglycemia-driven enhancement of channel trafficking to the neuronal plasma membrane. At the same time, the activation of the neuronal activity-dependent CaMKII signal transduction pathways as well as dysregulation of redox balance may contribute to the changes in the gating properties of T-type channels leading to their upregulation near resting potential under CFA-induced inflammation.

## Author's Note

In this study, the electrophysiological isolation of a specific subtype of rat nociceptive DRG neurons allowed to establish significant abnormalities in the functioning of voltage-gated channels and ASICs under conditions of peripheral inflammation. Functionally, these neurons of rats with CFA-induced peripheral inflammation demonstrate the sensitization to protons and increased excitability that may contribute to hyperalgesia and chronic inflammatory pain. Thus, this work contributes to a better understanding of the mechanisms of sensory neurons sensitization under the peripheral inflammation and the development of strategies to fight chronic inflammatory pain.

## Data Availability Statement

The original contributions presented in the study are included in the article/supplementary material, further inquiries can be directed to the corresponding author/s.

## Ethics Statement

The animal study was reviewed and approved by Animal Care and Use Committee at Bogomoletz Institute of Physiology, National Academy of Sciences of Ukraine.

## Author Contributions

DD, NV, and PB conceived the study, contributed to the analysis and interpretation of the data, and wrote and revised the manuscript. DD and NV obtained animals with CFA-induced inflammation in hind paws. DD performed the electrophysiological experiments. All authors have substantially contributed to the concept and design of the study, revision of the manuscript, and have read and approved the final version of the manuscript.

## Funding

This work was supported by the National Academy of Sciences of Ukraine grants Nos 0116U004470 and 0120U00 to PB and NV.

## Conflict of Interest

The authors declare that the research was conducted in the absence of any commercial or financial relationships that could be construed as a potential conflict of interest.

## Publisher's Note

All claims expressed in this article are solely those of the authors and do not necessarily represent those of their affiliated organizations, or those of the publisher, the editors and the reviewers. Any product that may be evaluated in this article, or claim that may be made by its manufacturer, is not guaranteed or endorsed by the publisher.
